# Fabrication of a Functionally Graded Copper-Zinc Sulfide Phosphor

**DOI:** 10.1038/srep23064

**Published:** 2016-03-14

**Authors:** Jehong Park, Kwangwon Park, Jongsu Kim, Yongseok Jeong, Akira Kawasaki, Hansang Kwon

**Affiliations:** 1 Next-Generation Materials Co., Ltd. (NGM), Building-7, 365, Sinseon-ro, Busan 48547, Korea; 2 Department of Display Engineering, Pukyong National University, Gaon-building, 905, Yongso-ro, Busan 48513, Korea; 3 Department of Materials Processing Engineering, Tohoku University, Sendai 980-8579, Japan; 4 Department of Materials System Engineering, Pukyong National University, Building-7, 365, Sinseon-ro, Busan 48547, Korea

## Abstract

Functionally graded materials (FGMs) are compositionally gradient materials. They can achieve the controlled distribution of the desired characteristics within the same bulk material. We describe a functionally graded (FG) metal-phosphor adapting the concept of the FGM; copper (Cu) is selected as a metal and Cu- and Cl-doped ZnS (ZnS:Cu,Cl) is selected as a phosphor and FG [Cu]-[ZnS:Cu,Cl] is fabricated by a very simple powder process. The FG [Cu]-[ZnS:Cu,Cl] reveals a dual-structured functional material composed of dense Cu and porous ZnS:Cu,Cl, which is completely combined through six graded mediating layers. The photoluminescence (PL) of FG [Cu]-[ZnS:Cu,Cl] is insensitive to temperature change. FG [Cu]-[ZnS:Cu,Cl] also exhibits diode characteristics and photo reactivity for 365 nm -UV light. Our FG metal-phosphor concept can pave the way to simplified manufacturing of low-cost and can be applied to various electronic devices.

Functionally graded materials (FGMs) are compositionally gradient materials with unique properties within the same bulk material. Compared to isotropic bulk materials, their compositions or microstructures gradually change over the volume. The desired properties of FGMs can be attained by controlling the distribution of the composition or microstructure in the gradient layers^1^,^2^,^3^,^4^,^5^,^6^,^7^,^8^,^9^. In mechanical parts that are composed of dissimilar materials, the concept of FGMs is of great advantage to enhance the durability of their components because gradient interfaces of FGMs can reduce thermal-mechanical stresses and mitigate delamination at crack-sensitive locations such as the interfaces of dissimilar materials^1^,^2^,^3^. The concept of FGMs has been implemented for various applications such as sensors, biomaterials, and functional metal composites because they can be designed to have unique functionality within the same bulk material compared to typical homogeneous bulk materials^4^,^5^,^6^,^7^,^8^,^9^.

Zinc sulfide (ZnS) is a well-known II–VI group semiconductor with a bandgap energy of 3.7 eV at room temperature. ZnS is one of the attractive materials in optoelectronic devices with applications such as field emitter, laser diode, and sensor^10^,^11^,^12^,^13^,^14^,^15^,^16^,^17^. Additionally, ZnS doped with impurities has been used as a traditional phosphor for light-emitting materials in display devices such as electroluminescence devices (ELDs), cathode-ray tubes (CRTs), and field emission devices (FEDs); for instance, Cu- and Cl-doped ZnS (ZnS:Cu,Cl) is an efficient phosphor emitting in the blue-green visible range^18^,^19^,^20^,^21^,^22^,^23^.

Recently, nanostructure (NS)-based ZnS has been identified as a high potential candidate for use in photo-sensing materials. However, most of the research on NS-based ZnS has been performed using inefficient methods involving time-consuming processes or complex chemical routes^12^,^13^,^14^,^15^,^16^,^17^. In addition, to fabricate functional devices, the materials must be appropriately processed for their functionality. Furthermore, to minimize the mismatch of physical properties between dissimilar materials, the processes can become even more complicated. In particular, conductive materials used as electrodes are indispensable in functional devices. To ensure long-term device reliability, the electrode must be tightly bonded, minimizing the mismatch of the physical coefficients (thermal expansion, thermal conductivity and electrical conductivity) with other functional materials constituting the functional device.

We report functionally graded (FG) metal-phosphor fabricated by a very simple powder process using a spark plasma sintering (SPS) technique^24^. In this research, we employed copper (Cu) and Cu- and Cl-doped ZnS (ZnS:Cu,Cl) as metal and phosphor materials, respectively. Cu and ZnS:Cu,Cl were successfully combined by adapting the concept of the FGM. The fabricated FG [Cu]-[ZnS:Cu,Cl] showed that one side had the intrinsic properties of an electrode and other side had the intrinsic properties of a phosphor within the same bulk material. To the best of our knowledge, this is the first structure that combines metal and phosphor as an adaptation of the FGM concept. In this paper, we investigate the luminescent properties and reactivity for ultraviolet (UV) -light as well as the morphology of the FG [Cu]-[ZnS:Cu,Cl].

## Results

**Figure 1:**
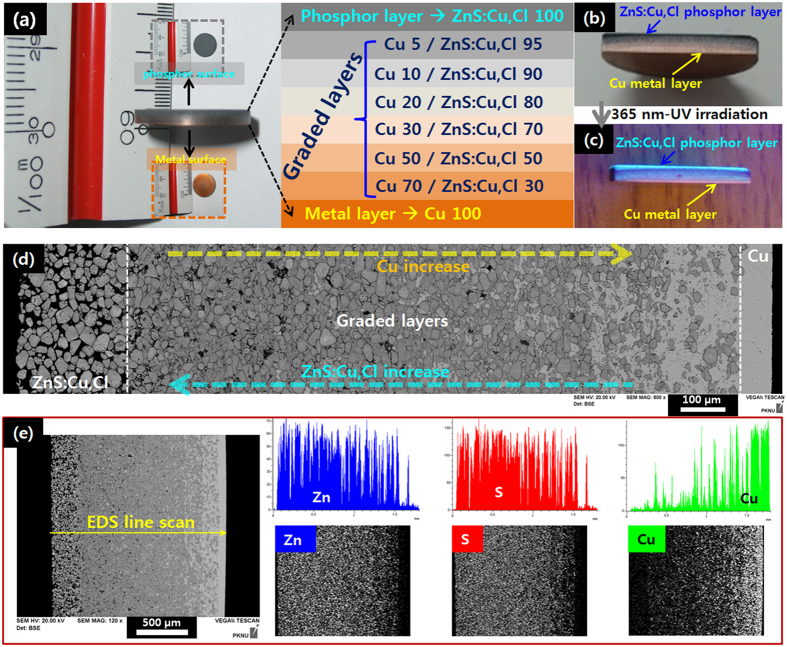
(**a**) Photograph and schematic diagram of FG [Cu]-[ZnS:Cu,Cl]. (**b**) Cross-sectional photograph of FG [Cu]-[ZnS:Cu,Cl]. (**c**) Cross-sectional photograph of FG [Cu]-[ZnS:Cu,Cl] under 365 nm -UV lamp. (**d**) Cross-sectional SEM image of FG [Cu]-[ZnS:Cu,Cl]. (**e**) Cross-sectional SEM image and EDS line scan (right upper), elemental mapping (right bottom) of FG [Cu]-[ZnS:Cu,Cl].

**Figure 2:**
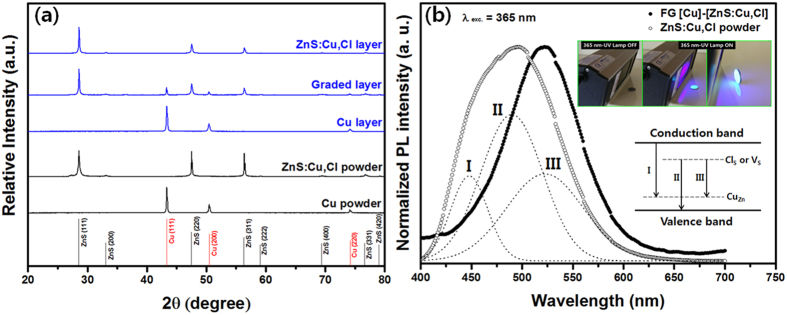
(**a**) XRD patterns of ZnS:Cu,Cl, graded, Cu layers in FG [Cu]-[ZnS:Cu,Cl] and primary Cu, ZnS:Cu,Cl powders. (**b**) PL spectra of FG [Cu]-[ZnS:Cu,Cl] and ZnS:Cu,Cl powder excited by 365 nm; insets are photographs of FG [Cu]-[ZnS:Cu,Cl] emitting under a 365 nm- UV lamp (upper) and energy levels within the band-gap of ZnS for dopants and acceptors (bottom).

**Figure 3:**
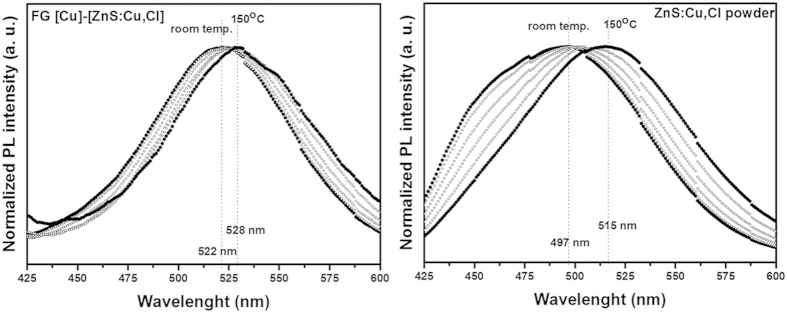
Normalized PL spectra of FG [Cu]-[ZnS:Cu,Cl] and ZnS:Cu,Cl powder as a function of temperature.

**Figure 4:**
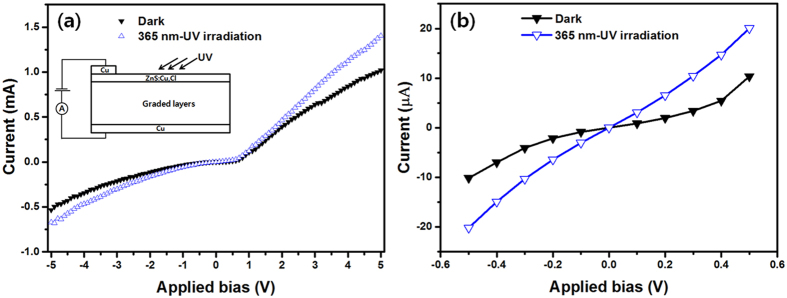
(**a**) *I–V* curves of FG [Cu]-[ZnS:Cu,Cl] in the dark and under 365 nm UV-lamp irradiation; the inset is a schematic diagram of the FG [Cu]-[ZnS:Cu,Cl] UV-light sensor configuration. (**b**) *I–V* curves of FG [Cu]-[ZnS:Cu,Cl] in the dark and under 365 nm UV-lamp irradiation [enlarged origin of (**a**)].

Functionally graded (FG) metal (Cu)-phosphor (ZnS:Cu,Cl) was fabricated by using a very simple powder process. Our FG [Cu]-[ZnS:Cu,Cl] revealed dual-structured functional materials composed of dense Cu and porous ZnS:Cu,Cl which were completely combined through six graded mediating layers and bluish-green light was emitted under a 365-nm UV lamp in the layer of ZnS:Cu,Cl (Figs 1 and 2). In the photoluminescence (PL) study, our FG [Cu]-[ZnS:Cu,Cl] exhibited stability upon temperature change because the Cu intimately combined with the ZnS:Cu,Cl. Cu acts as a heat sink in dual-structured FG [Cu]-[ZnS:Cu,Cl] (Fig. 3). In addition, the FG [Cu]-[ZnS:Cu,Cl] exhibited diode characteristics and photo reactivity for 365 nm- UV light (Fig. 4).

## Discussion

Figure 1 illustrates the fabricated FG [Cu]-[ZnS:Cu,Cl].; Six layers containing Cu and ZnS:Cu,Cl were combined as a graded mediating layer between pure Cu and ZnS:Cu,Cl layers. The FG [Cu]-[ZnS:Cu,Cl] was approximately 1.7 mm thick and had a diameter of 15 mm. Figure 1(b) shows a cross-sectional photograph of the FG [Cu]-[ZnS:Cu,Cl] cut by a diamond saw. The cross section shows a clear surface that was not delaminated or cracked between layers while cutting. This is evidence that the Cu and ZnS:Cu,Cl layers were completely combined. The Cu and ZnS:Cu,Cl layers can be obviously identified when 365 nm- ultraviolet (UV) light was irradiated on the cross-section of the specimen [Fig. 1(c), movies S1 and S2]. We confirmed the cross-sectional morphology of the FG [Cu]-[ZnS:Cu,Cl] using scanning electron microscopy (SEM). Figure 1(d,e) show cross-sectional SEM images of the FG [Cu]-[ZnS:Cu,Cl] and its elemental distribution data obtained by energy dispersive spectroscopy (EDS), respectively. Figure S1 in supplementary information (SI) shows segment images of the cross-sectional SEM image of Fig. 1(d). Figure 1(d) was composed by aligning the segment images of Fig. S1. The thickness of the Cu and ZnS:Cu,Cl layers in FG [Cu]-[ZnS:Cu,Cl] were found to be approximately 70 μm and 200 μm, respectively. The layers completely combined with the mediating graded layers (SEM images of the mixture powders composing the graded layers are presented in Figs S2 and S3 of SI). Additionally, we can confirm that the density of the Cu layer is higher than that of the ZnS:Cu,Cl layer. We expect that this dual structure of porous-dense material has an advantage in sensor applications^25^,^26^. In our case, the ZnS:Cu,Cl layer functions as the active layer detecting a change in the environment (absorption of photons or gas^12^,^13^,^14^,^15^,^16^,^17^), the porous structure of the ZnS:Cu,Cl expose more active sites to the environment than a dense structure would, and the Cu layer functions as an electrode that transfers electrons captured by the active sites of ZnS:Cu,Cl; the dense structure of the Cu provides more electron mobility than a porous structure would.

From the results of the EDS line scan and elemental mapping [Fig. 1(e)], we confirmed that Zn and S gradually decreased while Cu gradually increased moving towards the Cu layer. Additionally, a small Cu signal was detected in the ZnS:Cu,Cl layer, while small Zn and S signals were detected in the Cu layer, due to the inter-diffusion of Zn, S and Cu during the SPS process.

Figure 2(a) shows X-ray diffraction (XRD) patterns for the primary Cu and ZnS:Cu,Cl powders, and the graded layer, Cu and ZnS:Cu,Cl layers in FG [Cu]-[ZnS:Cu,Cl]. The reflection peaks of the Cu and ZnS:Cu,Cl powders were indexed to the cubic phase matched with JCPDS card no. 77–2100 (ZnS) and 70–3039 (Cu), respectively; no impurity phases were observed. All of the reflection peaks of the FG [Cu]-[ZnS:Cu,Cl] were well matched with the primary powders, and minor phase was slightly observed in graded layer; the XRD pattern of the graded layer revealed a bi-phase mixture of ZnS and Cu including slight third phase (Fig. S4). Interestingly, the XRD patterns of the ZnS in the ZnS:Cu,Cl and the graded layers were highly oriented to the (111)-crystal plane compared with the patterns of the ZnS:Cu,Cl powder. This is due to the effect of uniaxial pressure, where randomly oriented grains are re-arranged as a result of the pressure applied during the SPS process, thus graining a preferred orientation after SPS^27^. Figure S5(a,b) (SI) show enlarged regions of the (111)-crystal plane of ZnS and Cu, respectively. In the FG [Cu]-[ZnS:Cu,Cl], the reflection-peak positions for ZnS and Cu were slightly shifted towards higher and lower angles, respectively, compared with the peaks for the powders. This shift means that the crystal lattices of ZnS and Cu in the FG [Cu]-[ZnS:Cu,Cl] were slightly distorted, which is caused by inter-diffusion between Zn and Cu. The shift towards a large angle for ZnS is attributed to the contraction of the ZnS crystal lattice because of substitution by smaller Cu (1.28 Å) on Zn (1.34 Å) sites. Conversely, the shift towards a lower angle for Cu is attributed to the expansion of the Cu crystal lattice because of the substitution by larger Zn on Cu sites. These results are consistent with EDS results [Fig. 1(e)]. In addition, the reflection-peak position of ZnS in the ZnS:Cu,Cl layer shifted to a slightly larger angle than that of the graded layer [Fig. S5(a)]. We assume that this small shift is caused by sublimation of volatile S in the ZnS crystals during the SPS process, resulting in structural defects in ZnS caused by S vacancies that can induce lattice distortion.

Figure 2(b) shows the photoluminescence (PL) spectra of the FG [Cu]-[ZnS:Cu,Cl] and ZnS:Cu,Cl powder. The insets show photographs of FG [Cu]-[ZnS:Cu,Cl] emitting (bluish green) under a 365-nm UV lamp and the energy levels within the bandgap of ZnS. The PL spectrum of the ZnS:Cu,Cl powder shows a non-Gaussian distribution, consisting of I, II, III emission bands peaking at 448, 490 and 522 nm, respectively. Band I is ascribed to the electronic transition from the conduction band (CB) of ZnS to a Cu-substituted Zn site (Cu_Zn_) in ZnS. Band II is ascribed to the electronic transition from a Cl-substituted S site (Cl_S_) in ZnS to the valence band (VB) of ZnS. Band III is caused by donor (Cl)-acceptor (Cu) recombination. The PL spectrum of FG [Cu]-[ZnS:Cu,Cl] revealed different spectral distributions compared with that of ZnS:Cu,Cl powder. We can observe band III dominating the PL spectrum in FG [Cu]-[ZnS:Cu,Cl]. This is attributed to an increase in the concentration of Cu_Zn_ and V_S_ in ZnS in accordance with the XRD results in Fig. S5. We assume that excited electrons are trapped at V_S_ sites and Cl_S_ sites, and that these trapped electrons recombine at Cu_Zn_ sites.

Additionally, we investigated the PL properties of FG [Cu]-[ZnS:Cu,Cl] and ZnS:Cu,Cl powder as a function of temperature (Fig. 3). Table 1 presents the PL-peak position of FG [Cu]-[ZnS:Cu,Cl] and ZnS:Cu,Cl powder attained from the PL spectra in Fig. 3. In phosphors, a change of the PL -peak position with temperature is caused by electron-phonon interaction^23^. In our research, the PL-peak position of FG [Cu]-[ZnS:Cu,Cl] was less red-shifted than that of the ZnS:Cu,Cl powder as the temperature increased, which means that FG [Cu]-[ZnS:Cu,Cl] is less sensitive to temperature change. This behaviour can be explained by considering that residual heat in ZnS:Cu,Cl can escape more easily because of the high thermal conductivity of Cu (397 W m^−1^K^−1^) in FG [Cu]-[ZnS:Cu,Cl], because ZnS:Cu,Cl was intimately combined with Cu as seen in Fig. 1. Namely, Cu suppresses the generation of phonons in ZnS:Cu,Cl.

Finally, we tested the current(*I*)-voltage(*V*) curves for the FG [Cu]-[ZnS:Cu,Cl] in the dark and under 365 nm -UV irradiation (Fig. 4). These curves reveal asymmetric and nonlinear behaviour that resembles a Schottky diode. We cautiously assume that this behaviour is due to the graded layer of FG [Cu]-[ZnS:Cu,Cl] having graded electrical conductivity according to the Cu content. We speculate that the graded layer can affect the electron mobility under forward and reverse bias; a detailed study of the *I-V* characteristics is currently in progress. In any case, when the FG [Cu]-[ZnS:Cu,Cl] was irradiated by 365 nm -UV light, the current increased from 1.02 mA (dark condition) to 1.40 mA at an applied bias of 5 V [Fig. 4(a)] and the current increased from 10.34 μA (dark condition) to 20.05 μA at an applied bias of 0.5 V [Fig. 4(b)]. From these results, we conclude that our FG [Cu]-[ZnS:Cu,Cl] demonstrates reactivity for UV-light and that more quantitative study is needed to understand its performance in terms of photosensitivity, efficiency, and wavelength-dependent responsivity.

In summary, we successfully fabricated a novel functionally graded (FG) metal (Cu)-phosphor (ZnS:Cu,Cl) using a very simple powder process. Our FG [Cu]-[ZnS:Cu,Cl] revealed dual-structured functional materials composed of dense Cu and porous ZnS:Cu,Cl, which were completely combined through six graded mediating layers. In the photoluminescence (PL) study, our FG [Cu]-[ZnS:Cu,Cl] exhibited stability upon temperature change because the Cu intimately combined with the ZnS:Cu,Cl. Cu acts as a heat sink in dual-structured FG [Cu]-[ZnS:Cu,Cl]. In addition, the FG [Cu]-[ZnS:Cu,Cl] exhibited diode characteristics and photo reactivity for 365 nm- UV light. A detailed study of the *I–V* characteristics related to the unique structure of our FG [Cu]-[ZnS:Cu,Cl] is still underway. We expect that the concept of FG metal-phosphor can be applied to various metal and phosphor materials and that dual-structured FG metal-phosphors can be applied to various electronic devices such as solar cells and electroluminescence devices as well as sensors.

## Methods

Fabrication of functionally graded (FG) [Cu]-[ZnS:Cu,Cl] was carried out using the following simple powder process.

High-purity commercial Cu powder (dendrite, 99.99% ) and commercial Cu- and Cl-doped ZnS (ZnS:Cu,Cl, cubic, ~50 μm) were used as a raw materials. Mixtures of Cu and ZnS:Cu,Cl powders containing 5, 10, 20, 30, 50, 70 vol.% Cu in ZnS:Cu,Cl were prepared using a simple ball milling process for 30 min at 200 rpm in air. The pure Cu and ZnS:Cu,Cl powders were not ball-milled. Pure Cu, mixtures of Cu-ZnS:Cu,Cl, and pure ZnS:Cu,Cl powders were stacked layer by layer into a graphite mould with a diameter of 15 mm; 0.2 g of powder was used for each layer. Then the stacked powders were sintered using a customized spark plasma sintering (SPS) system (Fuji Electronic Industrials Co., Ltd., SPS-321Lx, Japan). The SPS was carried out at 900 °C (heating rate; 100 °C/min) for 5 min of 50 MPa of pressure. The fabricated FG [Cu]-[ZnS:Cu,Cl] was shaped as a round disk with a diameter of 15 mm and a thickness of approximately 1.7 mm, as shown in Fig. 1.

The morphology and structures of fabricated FG [Cu]-[ZnS:Cu,Cl] were analysed by using a scanning electron microscope (SEM, Tescan, Vega, Czech) equipped with an energy-dispersive spectrometer (EDS, Horiba, Emax, Japan) and an X-ray diffractometer (XRD, Rigaku, Ultima, Japan). The XRD operated at 40 kV and 40 mA with Cu Kα radiation. The photoluminescence (PL) spectra of fabricated FG [Cu]-[ZnS:Cu,Cl] and ZnS:Cu,Cl powder were obtained with a Darsa pro-5200 system (PSI, Korea) equipped with a temperature sensor using an excitation wavelength of 365 nm from a xenon lamp. In the case of ZnS:Cu,Cl powder, the powder was pressed into a disk with a diameter of 15 mm and a thickness of approximately 1.7 mm before the PL measurement. Current-voltage (*I–V*) curves of the FG [Cu]-[ZnS:Cu,Cl] were obtained using a Keithley 2400, and a 365 nm-UV source (UVItec Ltd, LF-204.LS, 4W, UK) was used for irradiation at distance of 10 cm between source and sample.

## Additional Information

**How to cite this article**: Park, J. *et al.* Fabrication of a Functionally Graded Copper-Zinc Sulfide Phosphor. *Sci. Rep.* **6**, 23064; doi: 10.1038/srep23064 (2016).

## Supplementary Material

Supplementary Movie S1

Supplementary Movie S2

Supplementary Information

## Figures and Tables

**Table 1:** PL-peak positions of FG [Cu]-[ZnS:Cu,Cl] and ZnS:Cu,Cl powder as a function of temperature (obtained from PL spectra of Fig. 3).

Temperature [°C]	PL-peak position of FG [Cu]-[ZnS:Cu,Cl]	PL-peak position of FG [Cu]-[ZnS:Cu,Cl]
Room temp.	522 nm	497 nm
50	522 nm	497 nm
75	523 nm	501 nm
100	528 nm	503 nm
125	530 nm	505 nm
150	528 nm	515 nm
