# Supplementary material for: Fabrication of a Functionally Graded Copper-Zinc Sulfide Phosphor
Source: Sci Rep. 2016 Mar 14;6:23064. doi: 10.1038/srep23064 (PMC4789794; doi:10.1038/srep23064)
Supplement: Supplementary Information [file srep23064-s3.doc]

Supplementary Information

Title: Fabrication of Functionally Graded Copper-Zinc Sulfide Phosphor

**Authors**:

Jehong Park^1^, Kwangwon Park^2^, Jongsu Kim^2^, Yongseok Jeong^2^, Akira Kawasaki^3^,

Hansang Kwon^1,4^,*

**Affiliations:**

^1^Next-Generation Materials Co., Ltd. (NGM), Building-7, 365, Sinseon-ro, Busan 48547, Korea.

^2^Department of Display Engineering, Pukyong National University, Gaon-building, 905, Yongso-ro, Busan 48513, Korea.

^3^Department of Materials Processing Engineering, Tohoku University, Sendai 980-8579, Japan.

^4^Department of Materials System Engineering, Pukyong National University, Building-7, 365, Sinseon-ro, Busan 48547, Korea.

*Correspondence to: E-mail: [kwon13@pknu.ac.kr](mailto:kwon13@pknu.ac.kr) (H. K.).

**Figure S1** shows segment images of the cross-sectional SEM image of figure 1(d). In the Cu- rich region of figure S1(d), ZnS:Cu,Cl particles were enclosed by Cu. In the ZnS:Cu,Cl-rich regions of figure S1(a), (b) and (c) (yellow arrows indicate Cu and red arrows indicate melt-phase ZnS:Cu,Cl), the pores between the ZnS:Cu,Cl particles were filled with Cu and melt-phase ZnS:Cu,Cl. The appearance of the melt-phase ZnS:Cu,Cl may be due to the very small ZnS:Cu,Cl particles crushed during the ball milling process. The crushed small particles have high surface energy and liquidity, allowing them to slide towards the pores where they can aggregate and melt at pore sites during the SPS process.

**Figure S2** and **S3** show SEM images of the powders after the ball-milling process; pure -ZnS:Cu,Cl and -Cu powders were not milled. As the Cu content of the mixtures increased, the ZnS:Cu,Cl particles were increasingly crushed to small particles during the milling process. This can be explained by Cu acting as a lubricant, reducing friction between the ZnS:Cu,Cl particles during the ball milling process. **Figure S3** reveals SEM and EDS elemental maps of Cu 50 vol.% / ZnS:Cu,Cl 50 vol.% and Cu 5 vol.% / ZnS:Cu,Cl 95 vol.%. The ZnS:Cu,Cl particles are more crushed to small particles in Cu 5 vol.% / ZnS:Cu,Cl 95 vol.%. We confirmed that in EDS mapping image, the crushed small particles are ZnS:Cu,Cl, not Cu.

**Figure S1**. Cross-sectional SEM images of FG [Cu]-[ZnS:Cu,Cl]; (a), (b), and (c) are ZnS:Cu,Cl-rich regions, and (d) is a Cu-rich region; the red and yellow arrows indicate melt -phases of ZnS:Cu,Cl and Cu, respectively. The green rectangles in each image indicate the same regions; figure 1d shows the overlapped green rectangles of each image in the order (a), (b), (c) and (d).


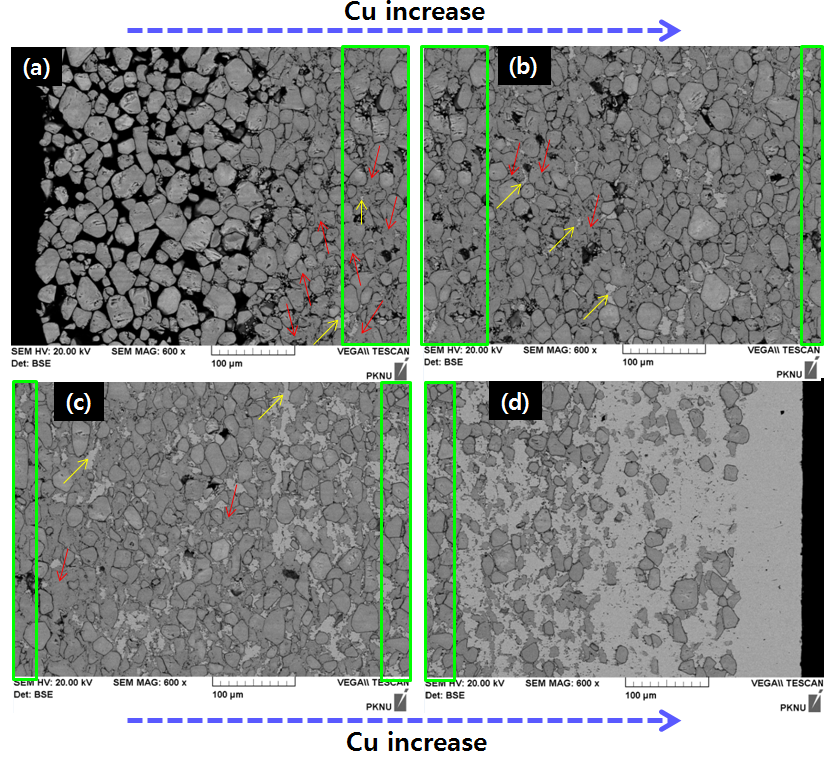


**Figure S2**. SEM images of primary Cu, ZnS:Cu,Cl and their mixture powders.


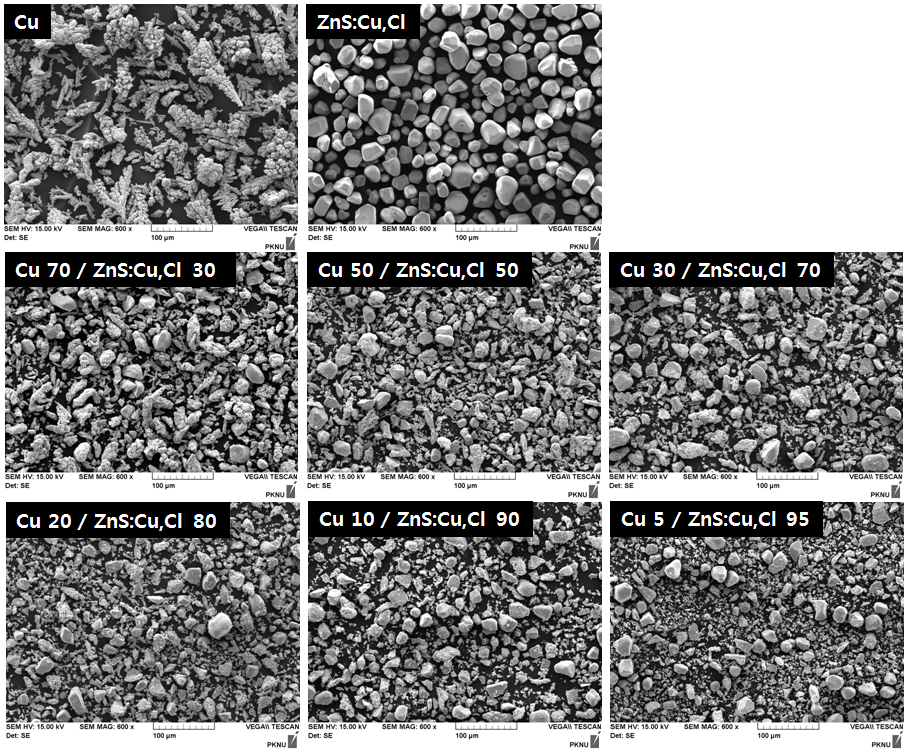


**Figure S3**. SEM images of (a) Cu 50 vol.% / ZnS:Cu,Cl 50 vol.% and (b) Cu 5 vol.% / ZnS:Cu,Cl 95 vol.%. powders and their EDS elemental maps.


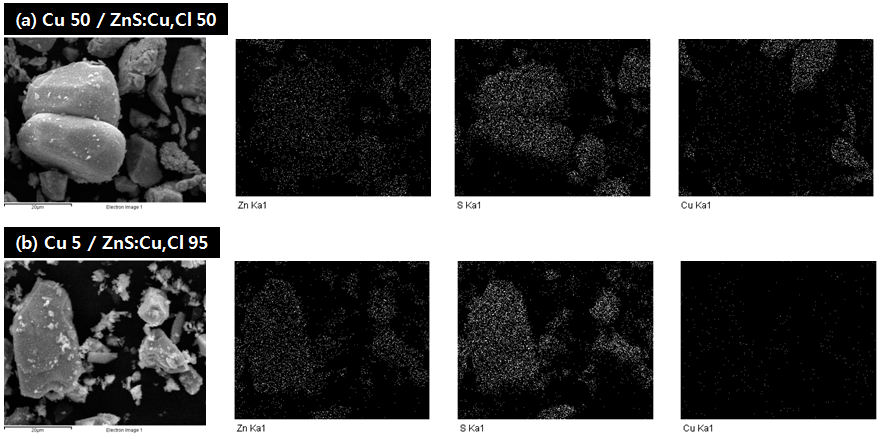


**Figure S4**. XRD pattern of graded layers in FG [Cu]-[ZnS:Cu,Cl] (selected from origin of figure 2(a)).


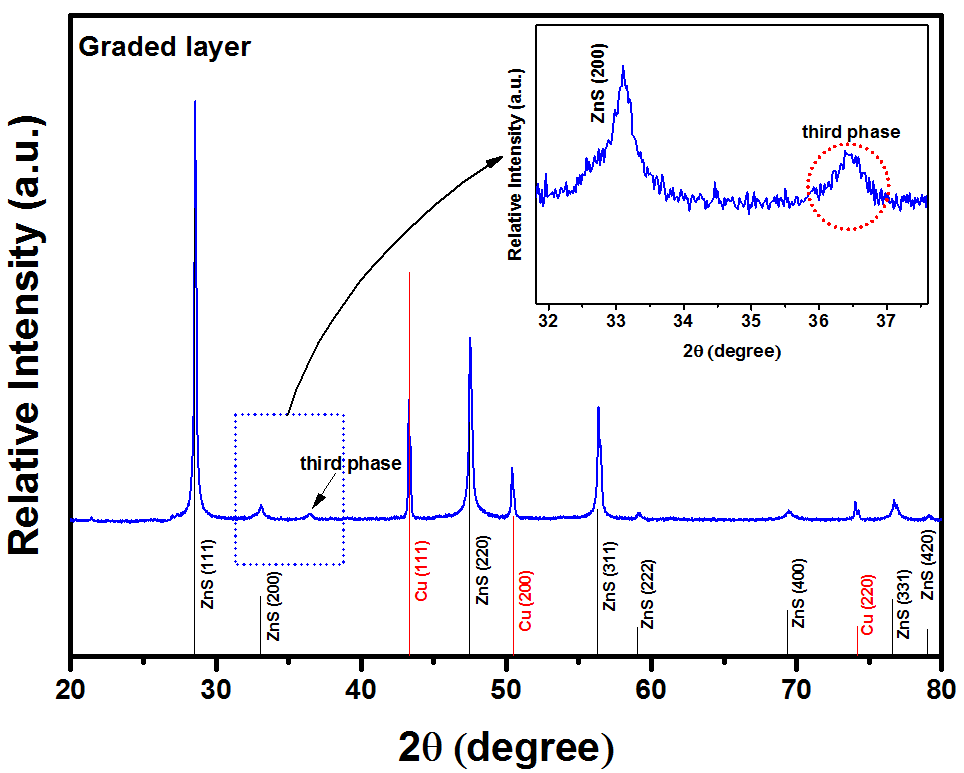


**Figure S5**. XRD patterns of (a) ZnS:Cu,Cl and graded layers in FG [Cu]-[ZnS:Cu,Cl] and primary ZnS:Cu,Cl powder and (b) Cu and graded layers in FG [Cu]-[ZnS:Cu,Cl] and primary Cu powder (enlarged origin of figure 2(a)).


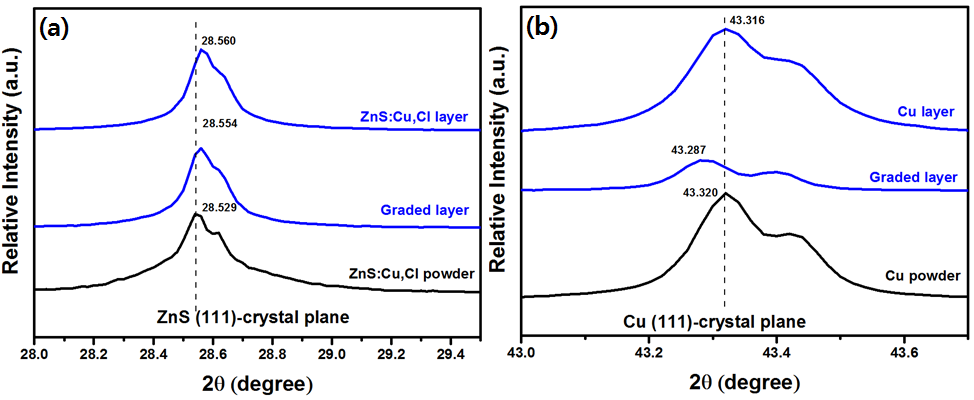


**Movie S1**. FG [Cu]-[ZnS:Cu,Cl]

**Movie S2**. Cross section of FG [Cu]-[ZnS:Cu,Cl]
